# Analysis of Genetic Diversity and Structure of Eight Populations of *Nerita yoldii* along the Coast of China Based on Mitochondrial *COI* Gene

**DOI:** 10.3390/ani14050718

**Published:** 2024-02-25

**Authors:** Senping Jiang, Zhenhua Li, Jiji Li, Kaida Xu, Yingying Ye

**Affiliations:** 1 National Engineering Research Center for Marine Aquaculture, Zhejiang Ocean University, Zhoushan 316022, China; jiangsenping@zjou.edu.cn (S.J.); lijiji@zjou.edu.cn (J.L.); 2 Key Laboratory of Sustainable Utilization of Technology Research for Fisheries Resources of Zhejiang Province, Scientific Observing and Experimental Station of Fishery Resources for Key Fishing Grounds, Ministry of Agriculture and Rural Affairs of China, Zhejiang Marine Fisheries Research Institute, Zhoushan 316021, China; lzh0580@zjou.edu.cn

**Keywords:** *Nerita yoldii*, mitochondrial *COI*, genetic diversity, genetic structure

## Abstract

**Simple Summary:**

The mitochondrial *COI* gene as a molecular marker is widely used in genetic diversity and population genetic diversity analysis. In this study, mitochondrial *COI* was used to analyze the genetic diversities of eight populations of *Nerita yoldii* along the coast of China. The results showed that *N. yoldii* has a high level of genetic diversity, with genetic structure differences observed between the Xiapu population and six other populations, excluding the Taizhou population. The results of neutral tests and nucleotide mismatch analyses indicate a historical demographic expansion in the *N. yoldii* population. Our study results provide foundational data for the conservation and sustainable utilization of *N. yoldii* resources.

**Abstract:**

*Nerita yoldii* is a euryhaline species commonly found in the intertidal zone. To investigate the genetic diversity of 233 *N. yoldii* individuals from eight locations along the coast of China, we utilized the mitochondrial *COI* gene as a molecular marker. A total of 34 haplotypes were detected, exhibiting a mean haplotype diversity (*Hd*) of 0.5915 and a mean nucleotide diversity (*Pi*) of 0.0025, indicating high levels of genetic diversity among all populations. An analysis of molecular variance (AMOVA) indicated that the primary source of genetic variation occurs within populations. In addition, neutral tests and mismatch analyses suggested that *N. yoldii* populations may have experienced bottleneck events. Moderate genetic differentiation was observed between Xiapu and other populations, excluding the Taizhou population, and may be attributed to the ocean currents. Intensively studying the genetic variation and population structure of *N. yoldii* populations contributes to understanding the current population genetics of *N. yoldii* in the coastal regions of China. This not only provides a reference for the study of other organisms in the same region but also lays the foundation for the systematic evolution of the Neritidae family.

## 1. Introduction

The family Neritidae comprises more than 300 species and is recognized for its remarkable diversity and extensive geographical distribution. Fossil evidence suggests that their ancestors inhabited the late Cretaceous period [1]. The adaptability of these gastropods to different salinity levels and habitats makes them a widely distributed group that thrives in brackish, marine, and freshwater ecosystems. They mainly inhabit tropical to subtropical regions, with only a few species found in temperate regions [2], such as *Nerita atramentosa*, primarily distributed in the temperate regions of southern Queensland (eastern) and western Australia [3], and the Japanese species *Neritilia mimotoi*, which inhabits the warm temperate region [2]. A previous study shed light on the factors contributing to the broad salinity tolerance of Neritidae species, which can be attributed to their recurrent colonization of marine, brackish, and freshwater environments throughout their evolutionary history [4]. The life history of most species in the Neritidae family is not well-documented, but it is established that most of them undergo a relatively prolonged planktonic phase.

Belonging to the family Neritidae, *Nerita yoldii* Récluz 1841 exhibits an overall semi-oval shape, characterized by a row of fine teeth on the inner edge of the outer lip and two to three teeth in the center of the inner lip. Although its appearance is described in detail, it is difficult to distinguish it from other species in the family of Neritidae based on morphological characteristics alone. For example, due to the similar morphological characteristics, Tsuchiya (2017) [5] regards *N. yoldii* to be a synonym of *Nerita squamulata* Le Guillou, 1841 in a 2000 study. With the advancement of molecular biology techniques, molecular markers have become a commonly used auxiliary method for species identification, aiding in the differentiation of other species within the Neritidae family. In China, predominantly inhabiting the coastal regions south of Zhejiang Province in the 1960s [6], *N. yoldii* thrives in the intertidal reef hard-bottom areas. Temperature plays a pivotal role in shaping the distribution of *N. yoldii*. However, recent studies have detected signs of its presence beyond Zhejiang Province, specifically in areas north of the province, such as Jiangsu Province. The migration of *N. yoldii* across the Yangtze River barrier into the more northern Jiangsu Province is believed to be a consequence of climate change, particularly global warming and rising temperatures, alongside human activities. The spawning period of *N. yoldii* in China was April–August, and the pelagic larvae of *N. yoldii* from the pristine marginal seas south of the Yangtze River also provide the potential for it to overcome the Yangtze River barrier and contribute to genetic variation among populations [7,8].

Avise (2000) [9] and Hewitt (2000) [10] mentioned that the distribution and genetic structure of the marine animals were closely related to historical climatic changes in the sea level, water temperature, and ocean currents. Compared to terrestrial species, marine animals are generally considered to have no obvious genetic structure and low genetic diversity. Especially in some Mollusca with a planktonic period, their plankton, larval or adult, could be transmitted with the ocean current and usually do not have many physical barriers to movement [11]. Planktonic larvae spread with the ocean currents and influence genetic structure by facilitating the exchanges of gene flow within populations [12]. The length of the planktonic larvae also is a significant factor in their distribution and genetic structure, and longer planktonic larval durations confer greater dispersal ability [13,14,15]. For example, Ye (2018) [16] used the *COI* gene to explore the genetic diversity and genetic structure of the clam *Gomphina aequilatera* in China and found that the distribution and genetic structure of *G. aequilatera* were related to the ocean current; the study concluded that species with a short planktonic larval stage have a strong population structure.

Mitochondrial cytochrome coxidase (COX) is an important component of the fourth protein complex in the mitochondrial respiratory chain, and it plays a significant role in various physiological activities, especially in energy supply, apoptosis, and metabolism [17]. The mitochondrial *COI* gene has advantages, including high conservatism, a low mutation rate, and maternal inheritance [18,19,20]. Consequently, it has emerged as a widely utilized molecular marker in genetic studies of almost all groups of animals, protists, and plants, especially for investigating phylogeny and genealogical geography [21,22,23,24,25,26,27]. It also has been widely used in Mollusca, such as *Ruditapes philippinarum* [28], *Haliotis diversicolor* [29], *freshwater mussels* [30], and so on.

In this study, we focused on examining the genetic diversity, genetic structure, and population history dynamics of *N. yoldii* along the coast of China, utilizing the mitochondrial *COI* gene. Our objectives were to characterize the genetic diversity of *N. yoldii* within and between populations. The results from this study may provide some support for using the germplasm resources and maintaining the genetic resources of *N. yoldii*.

## 2. Materials and Methods

### 2.1. Experimental Animals and Sampling

**Figure 1:**
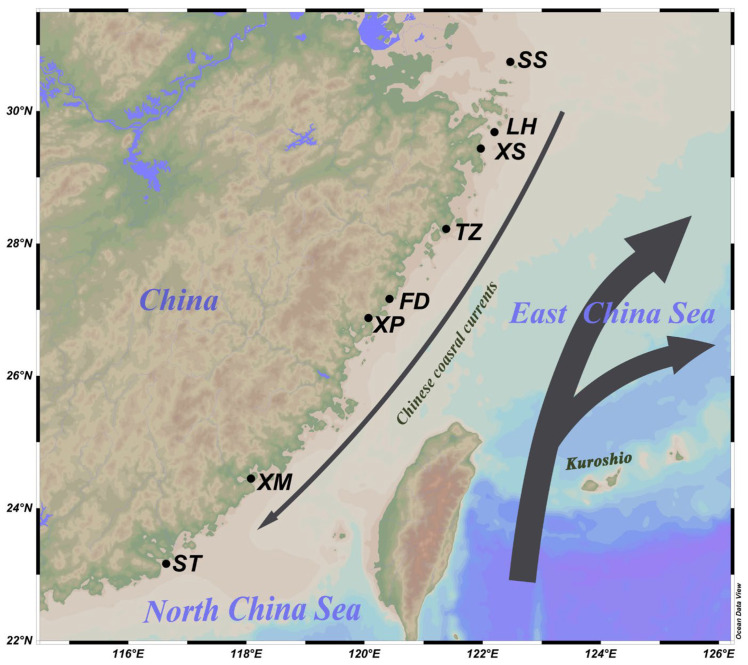
Picture showing the sampling locations along the coast of China. SS stands for Shengsi, LH stands for Liuheng, XS stands for Xiangshan, TZ stands for Taizhou, FD stands for Fuding, XP stands for Xiapu, XM stands for Xiamen, and ST stands for Shantou.

According to the *N. yoldii* geographical distribution along the coast of China, we randomly selected eight sites from the coast of China and collected a total of 233 individuals (Shengsi [SS], Liuheng [LH], Xiangshan [XM], Taizhou [TZ], Fuding [FD], Xiapu [XP], Xiamen (XM), Shantou [ST]) along the coast of China (Figure 1, Table 1). All experimental samples were approved by the State Oceanic Administration of China and the Ethics Committee of Zhejiang Ocean University and were performed according to national laws and regulations. The total samples were identified, and their muscle tissues were collected and saved in absolute alcohol at −20 °C.

### 2.2. DNA Extraction and Polymerase Chain Reaction Amplification

The genomic DNA of each sample was extracted by a salt-extraction procedure [31] and measured on a 1.5% agarose gel, then stored in a −20 °C freezer for later use. Based on the complete mitochondrial genome (MK395169) of *N. yoldii*, primers were designed using Primer Premier 5.0 software (Premier Biosoft International, San Franciso, CA, USA). The forward primers and reverse primers were 5′-GCACTAAGTGAGTCCTTGTTAAAT-3′ (COI-F) and 5′-ATTGATAGCCAAATCAAATTGTAAC-3′ (COI-R), respectively. The PCR reactions were performed in a total volume of 25 μL, comprising 12.5 μL of Taq MasterMix (Beijing Com Win Biotech Co., Ltd., Beijing, China), 1 μL of forward primer, 1 μL of reverse primer, 1 μL of genomic DNA, and 10 μL of ddH_2_O. The PCR amplification procedure was predenaturation at 95 °C for 3 min, then denaturation at 95 °C for 30 s, annealing at 49 °C for 30 s, 72 °C for 1 min for 30 cycles, and finally 72 °C for 10 min. The amplification products were detected and observed with the 1.5% agarose gel and sent to TSINGKE Biotech Co., Ltd. (Hangzhou, China) for bidirectional sequencing.

### 2.3. Data Analysis

The sequencing results were analyzed with BioEdit v7.2.6.1 software, and the fragments with standard peak type and no interference were screened and made into FASTA files with Microsoft Excel 2016 (MSO, Version 2312. The *COI* sequences of all sequenced *N. yoldii* individuals were serially compared using the BLAST tool in the NCBI database (URL: https://blast.ncbi.nlm.nih.gov/Blast.cgi, accessed on 21 May 2023) to confirm the species attribution of the sample, and individuals with a comparison similarity of less than 99% were eliminated and saved as FASTA files. MEGA-X software [32] was used to compare sequences and cut the ends flat. Then, DnaSP 6 software [33] was used to distinguish haplotypes and count the classical genetic parameters, such as haplotype diversity (*h*), nucleotide diversity (*Pi*), etc. It was also used in a mismatch analysis. An analysis of molecular variance (AMOVA), a genetic differentiation index (*F_ST_*), and a neutrality test between two populations were performed with Arlequin v3.5.2.2 software [34]. Network 10.2.0.0 software [35] was used to build a haplotype network diagram, and a phylogenetic tree was built with the final MEGA-X software. Gene flow network diagrams were constructed using divMigrate-online software [36].

## 3. Results

### 3.1. Characteristics of the COI Sequence and the Genetic Diversity of N. yoldii

The total length of the *COI* gene after comparison shearing was 494 bp, which was amplified from 233 individuals of *N. yoldii*. The average nucleotide frequencies among all sequences were 41.8% T, 14.2% C, 23.1% A, and 20.9% G, respectively. The A/T content (64.9%) was significantly higher than the C/G content (35.1%). Only 34 variable sites were found. Genetic diversity within the *N. yoldii* populations along the coast of China was investigated by analyzing the mitochondrial *COI* sequences (Table 2). Haplotype analysis showed a total of 34 haplotypes across the eight populations. Among the 233 *COI* sequences examined, haplotype numbers (*h*) ranged from 4 (LH) to 12 (ST). In this study, the haplotype diversity (*Hd*) across the eight populations exhibited a wide range, spanning from 0.2292 to 0.7536, with the majority of the populations displaying a pronounced high level of haplotype diversity (*Hd* > 0.5). Notably, the LH population stood out as an exception, demonstrating relatively lower haplotype diversity (*Hd* < 0.5). The nucleotide diversity (*Pi*) ranged from 0.0008 to 0.0037 across the eight populations, with all population exhibiting low levels of nucleotide diversity (*Pi*) (*Pi* < 0.005). Additionally, the average nucleotide difference (*K*) concurred with the nucleotide diversity (*Pi*), revealing minimal population-level differentiation.

### 3.2. Population Genetic Structure of N. yoldii

**Figure 2:**
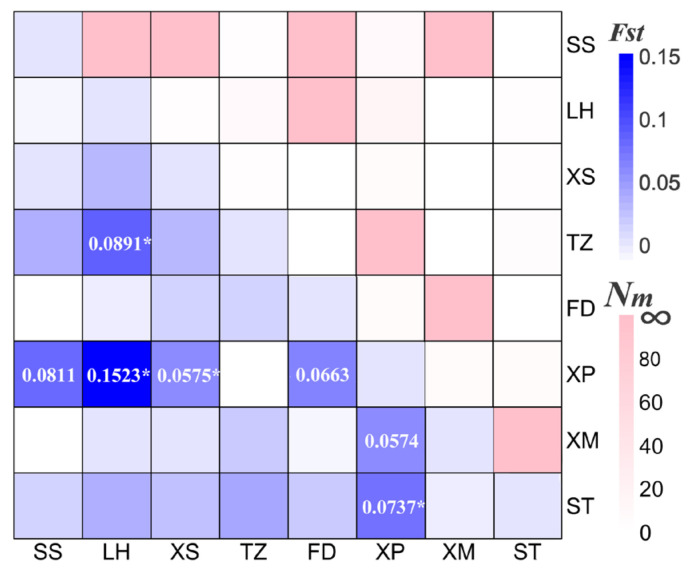
*F_ST_* and *Nm* of eight *N. yoldii* populations based on *COI* gene. Separated by the diagonal line, the blue part represents the *F_ST_* and the red part represents the gene flow, and the darker the color, the greater the value. ‘*’ indicates significance with probability at 0.05, and the *F_ST_* greater than 0.05 is displayed.

**Figure 3:**
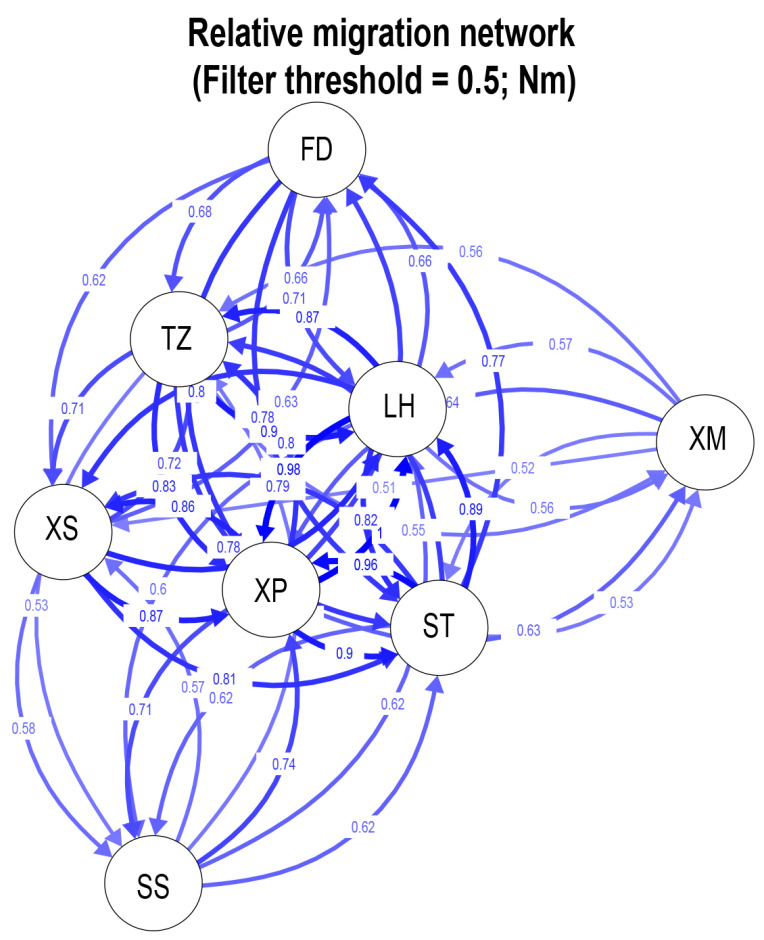
Directional relative migration networks of eight *N. yoldii* populations constructed with divMigrate using *Nm* values above 0.5.

**Figure 4:**
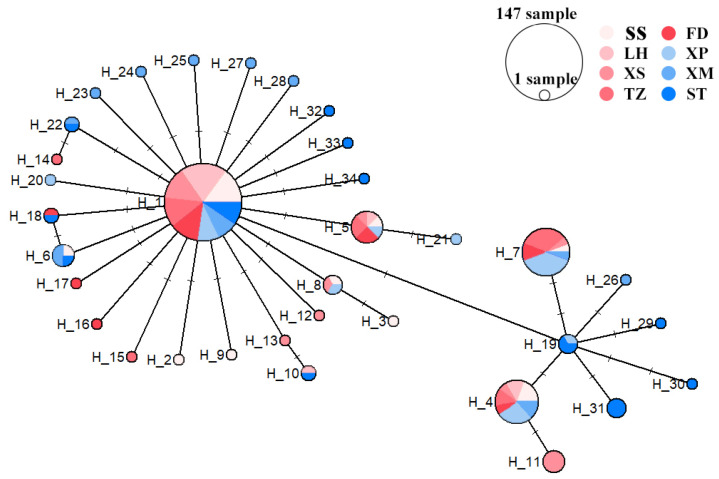
The haplotype network of eight *N. yoldii* populations based on *COI* gene. The colors represent the different populations, and the circle size represents the number of haplotypes.

**Figure 5:**
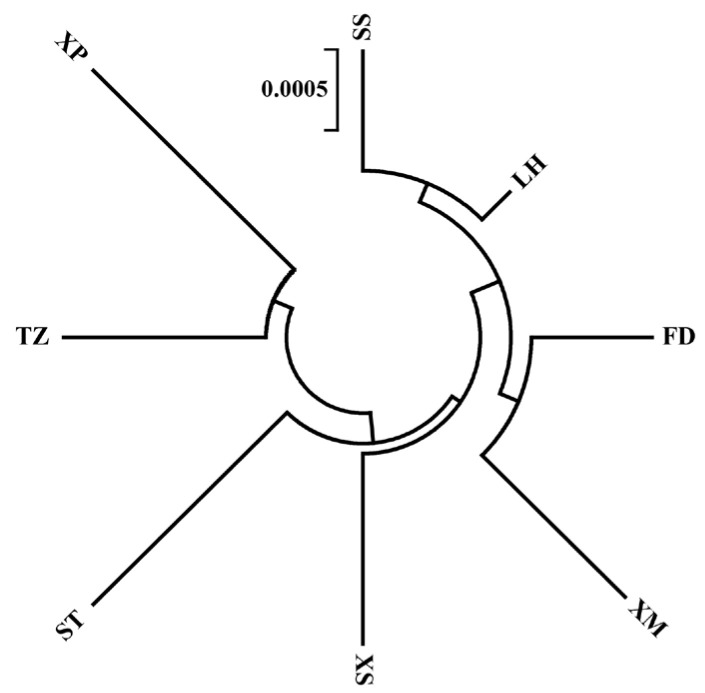
The neighbor-joining phylogenetic tree based on genetic distances of the mitochondrial *COI* gene of eight *N. yoldii* populations.

Genetic distances within populations ranged 0.0008 to 0.0037, while distances between populations ranged 0.0014 to 0.0036. The results of the molecular variance analysis (AMOVA) across the eight *N. yoldii* populations indicated that the primary genetic variations occurred within populations, representing 96.62% of the total variation. Contrary to the assumption of low levels of genetic variation among populations (3.38%), a significant portion of the variation was observed within populations (Table 3). The results of the pairwise *F_ST_* values between populations ranged from −0.0002 to 0.1523. While most results indicated no differentiations and lacked significance, medium levels of differentiation (0.05 < *F_ST_* < 0.25) were observed between the XP population and the other populations, except for the TZ population (Figure 2). Additionally, significant differentiations were evident between the XP population and the other four populations (SS, LH, XS, and ST). Migration dynamic analysis (Figure 3) revealed varying degrees of gene exchanges among different populations. The gene flow (Figure 2) confirmed a degree of genetic differentiation among populations. Furthermore, the gene flow between the XP population and the other populations was weak, except for the TZ population. The haplotype network illustrated a lack of significant differentiation among the 34 haplotypes in the eight populations along the Chinese coast, with XP population haplotypes concentrated on the right (Figure 4). Notably, haplotype 1 was located at the center, closely related to most other haplotypes, suggesting its ancestral nature within these *N. yoldii* populations. Conversely, haplotype 19 branched off separately, with most haplotypes within this branch distributed among the XP, XM, and ST populations. The phylogenetic tree constructed through the neighbor-joining analysis (Figure 5) revealed a close relationship between the XM and ST populations. Similarly, the LH population showed close proximity to the SS population. The XP population was closely related to the TZ population but distantly related to several other populations.

### 3.3. Population Phylogenetic Analysis

**Figure 6:**
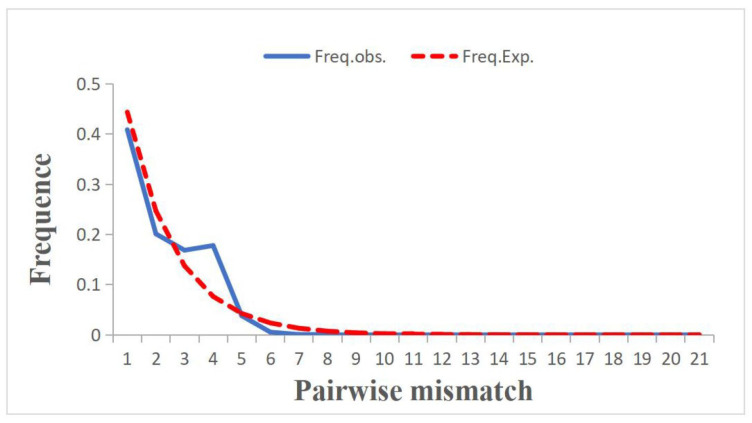
The mismatch analysis of eight *N. yoldii* populations based on *COI* gene.

In the Tajima’s D test, the test value of each population was found to be negative and significantly deviated from neutrality, except for the XS, TZ, and XP populations. Similar trends were observed in the Fu’s FS test, with the test value of each population displaying significant negative deviation from neutrality, except for the LH, TZ, and XP populations (Table 4). The results from both the Tajima’s D test and the Fu’s FS test suggest that *N. yoldii* might have been subjected to negative selection or bottleneck amplification during its evolutionary history. The mismatch analysis yielded results that differed from the observed values, indicating a unimodal Poisson distribution that closely resembled the expected curve, thus pointing towards an expansion effect within the population (Figure 6). By utilizing the mitochondrial evolution rate of the *N. yoldii* populations, it was estimated that the expansion of the cockle population occurred approximately during the Pleistocene.

## 4. Discussion

### 4.1. Genetic Diversity of the Eight Populations of N. yoldii along the Coast of China

Genetic diversity plays a crucial role in biological evolution as richer genetic diversity allows for greater adaptability to the environment and facilitates increased survivability. Grant (1998) [37] classified haplotype diversity levels and nucleotide diversity levels, considering haplotype diversity greater than 0.5 as high and vice versa. Similarly, nucleotide diversity greater than 0.005 is considered high and vice versa. Conversely, diminished genetic diversity often results in vulnerability to bottleneck effects and other factors that can lead to population decline. In the present study, the eight populations from the coast of China were marked by the *COI* gene. The mean *Hd* of these populations was 0.5915, indicating a high state, while the mean *Hd* was 1.2548 and the mean *Pi* was 0.0025, signifying a comparatively low state. The results of the mean *Hd* and *Pi* were similar to *Thais clavigera*, which was also analyzed using the *COI* gene. The mean *Hd* of the nine populations of *T. clavigera* distributed along the coast of China was 0.69826, slightly higher than the *N. yoldii* populations, and the mean *Pi* was 0.00182, slightly lower than the *N. yoldii* populations. However, both exhibited high levels of haplotype diversity and low levels of nucleotide diversity [38]. This situation can be interpreted as a bottleneck effect. The characteristics of a population undergoing a bottleneck include a significant reduction in population size at a specific period, leading to a small effective population size and a decline in genetic diversity. Subsequently, there is a rapid population expansion, facilitating the retention of new mutations and resulting in a recovery of genetic diversity [9,39]. Feng et al. (2021) [40] estimated that the divergence of *N. yoldii* occurred approximately 16.89 Mya, during the Neogene. This suggests that *N. yoldii* may have experienced the Quaternary glaciations. The East China Sea, situated in the western Pacific, had one of the most extensive shelves globally, exposing a total area of 850,000 km^2^ during the Pleistocene ice ages [41]. In this environmental context, the habitat of *N. yoldii* was constrained, leading to a sharp decline in population size, a reduction in effective population size, and a decrease in genetic diversity. As the Ice Age concluded, the habitat of *N. yoldii* recovered, and its population underwent rapid growth, promoting the retention of new mutations. Consequently, *N. yoldii* exhibited a condition of high haplotype diversity and low nucleotide diversity, a pattern observed in many marine invertebrates [42,43].

### 4.2. Population Differentiation of the Eight Populations of N. yoldii along the Coast of China

The measurement of *F_ST_* is critical in determining population differentiation in genetic studies. An *F_ST_* value ranging from 0 to 0.05 indicates no differentiation, 0.05 to 0.15 signifies a low level of differentiation, 0.15 to 0.25 suggests a medium level of differentiation, and values exceeding 0.25 imply a significant degree of differentiation [44]. In this study, the *F_ST_* value of the XP population and the other populations ranged from 0.05 to 0.15, except for the TZ population, with a significance level of *p* < 0.05, except for the TZ, FD, XP, and XM populations. These findings suggest a noticeable differentiation between the XP population and all the other populations, excluding the TZ population. We postulate that these differences may be associated with the Kuroshio Current and coastal currents [43,45]. Some marine organisms commonly show reduced genetic differentiation across extensive spatial ranges, displaying limited genetic structure. This phenomenon is often linked to the presence of a planktonic phase in many marine invertebrates, during which larvae disperse via ocean currents. This widespread dispersal mechanism facilitates gene flow among diverse geographic populations, contributing to lower levels of genetic differentiation and the manifestation of genetic homogeneity [46].

Originating between 12 and 15 degrees north latitude around the Philippines, the Kuroshio Current enters the East China Sea through the East Taiwan Channel, situated between the northeast of Taiwan and Yonaguni-Jima Island at the southwestern tip of the Ryukyu Islands. Additionally, smaller portions of the Kuroshio intrude into the East China Sea shelf through the Luzon and Taiwan Straits [47]. In addition, the coastal current is a main reason for the population differentiation. Especially, observations by Yuan and Li suggest a prominent southwestward stream of shelf water across the East China Sea that is discernible from the distribution of fine-grained sediments [48,49]. In all coastal currents, the Zhejiang–Fujian current exhibits distinct seasonal characteristics [50]. During the winter, under the action of northerly winds, the Yangtze River rushes southward along the Zhejiang coast to the Fujian coast, while in the summer, it flows northward along the coast [51]. Simultaneously, under the influence of robust northeast winds, the East Guangdong Sea Current travels southwest, intersecting with the Fujian and Zhejiang Sea Currents. In the summer, the East Guangdong Sea Current flows northeast, entering the Taiwan Strait [52,53]. Similar to many Mollusca [12], *N. yoldii* feature a planktonic phase. During this period, guided by ocean currents, frequent gene exchanges between populations occur, potentially accounting for the phenomenon of the *N. yoldii* differentiation.

### 4.3. Phylogeny of the Eight Populations of N. yoldii along the Coast of China

The phylogeny of the eight populations of *N. yoldii* along the coast of China is strongly influenced by the significant role of the China Southeast Sea as a key component of the northwest Pacific, representing a vital marine center of origin [54]. This sea is delineated into two marginal seas, the East China Sea and the South China Sea, separated by the Taiwan Strait. During the glacial epoch, the drop in sea levels facilitated the connection of the Taiwan Strait with the shelf of the East China Sea, leading to the physical segregation of the East China Sea and the South China Sea. Conversely, during the interglacial periods, the rise in sea levels caused the Taiwan Strait to disconnect from the shelf of the East China Sea, enabling the interconnection between the East China Sea and the South China Sea [54,55,56]. These geological phenomena provided the potential for historical population demographic expansion in the marine environment. The findings derived from the neutral tests and mismatch analyses conducted on *N. yoldii* substantiate the idea that similar evolutionary processes have shaped its genetic history. We estimated the population expansion time using the formula t = τ/(2μk), where τ represents the parameter in the sudden expansion model, k denotes the sequence length in our study, and μ is the mutation rate across the entire sequence [57]. Unfortunately, we could not determine a precise value for μ, so we conducted a simple estimation of the expansion time. The estimated expansion time falls approximately within the Pleistocene epoch [58]. This observation lends additional support to the notion that *N. yoldii* may have experienced the geological events mentioned earlier. It is speculated that *N. yoldii* may come from the same ancestral group from shelters in the East China Sea during the glacial period towards the end of the Pleistocene epoch. Additionally, the features presented by the haplotype network also corroborate this point. In this network, individual haplotypes primarily accumulate through gradual base mutations from the ancestral haplotype, showing an overall lack of distinct branches, with the majority of haplotypes clustering around the central ancestral haplotype.

## 5. Conclusions

This research utilized the mtDNA *COI* gene as a molecular marker to investigate the genetic diversity and structure of eight *N. yoldii* populations along the coast of China. The investigation revealed a substantial presence of high levels of genetic diversity and low levels of nucleotide diversity of *N. yoldii*. Generally, no significant genetic structures were observed, except for the XP population, which displayed marked and moderate genetic differentiation. The exception was the TZ population, possibly influenced by the Kuroshio Current and coastal currents. These findings enhance our understanding of the genetic diversities of *N. yoldii* and provide reference data for the rational and effective utilization of and research on the family Neritidae. Unfortunately, samples from the northernmost region (north of the Yangtze River) failed to be collected. Further research is needed to investigate whether the observed northward migration has genetic implications.

## Figures and Tables

**Table 1:** The sampling details for eight populations of *N. yoldii*.

Sampling Site	Abbreviation	Coordinates	Sampling Date	Sample Size
Shengsi	SS	30°44′ N 122°28′ E	2023.03	32
Liuheng	LH	29°40′ N 122°12′ E	2021.01	33
Xiangshan	XS	29°26′ N 121°58′ E	2021.01	30
Taizhou	TZ	28°13′ N 121°22′ E	2023.05	30
Fuding	FD	27°09′ N 120°25′ E	2019.08	26
Xiapu	XP	26°52′ N 120°04′ E	2018.11	30
Xiamen	XM	24°26′ N 118°04′ E	2021.04	24
Shantou	ST	23°09′ N 116°38′ E	2020.12	28
Total				233

**Table 2:** Genetic diversity analysis of eight *N. yoldii* populations based on *COI* gene.

Population	*S*	*h*	*Hd*	*K*	*Pi*	*PiJC*
SS	10	9	0.5282	0.9597	0.0019	0.0020
LH	5	4	0.2292	0.4167	0.0008	0.0009
XS	9	8	0.5494	1.2575	0.0026	0.0026
TZ	8	6	0.6092	1.5816	0.0032	0.0032
FD	8	7	0.5231	0.9631	0.0020	0.0020
XP	8	8	0.7287	1.8046	0.0037	0.0037
XM	13	11	0.7536	1.4420	0.0029	0.0029
ST	13	12	0.7487	1.4418	0.0029	0.0029
Total	34	34	0.5838	1.2334	0.0025	

*S*, mutation sites; *h*, haplotype numbers; *Hd*, haplotype diversity; *K*, average number of pairwise divergences; *Pi*, nucleotide diversity; *PiJC*, nucleotide diversity with JC.

**Table 3:** AMOVA analysis of eight *N. yoldii* populations based on *COI* gene.

Source of Variation	d.f.	Sum of Squares	Variance Components	Percentage of Variation
Among populations	7	8.593	0.02127 Va	3.38
Within populations	225	136.961	0.60872 Vb	96.62
Total	232	145.554	0.62999	

**Table 4:** The neutral test of eight *N. yoldii* populations based on *COI* gene.

Statistics	SS	LH	XS	TZ	FD	XP	XM	ST	Mean	s.d.
Tajima’s D	−1.92	−1.76	−1.39	−0.66	−1.70	−0.32	−2.03	−1.90	−1.46	0.63
Tajima’s D *p*-value	0.01	0.01	0.07	0.28	0.03	0.42	0.01	0.01	0.10	0.16
FS	−5.40	−1.55	−3.00	−0.30	−3.14	−1.65	−7.16	−8.07	−3.78	2.80
FS *p*-value	0.00	0.06	0.03	0.46	0.01	0.19	0.00	0.00	0.09	0.16

## Data Availability

Data are contained within the article and Supplementary Materials.

## Supplementary Materials

The following supporting information can be downloaded at https://www.mdpi.com/article/10.3390/ani14050718/s1, Table S1: The 34 haplotypes distribution of eight *N. yoldii* populations based on *COI* gene.; Table S2: Genetic distance of eight *N. yoldii* populations based on *COI* gene.
