# Supplementary material for: Analysis of Genetic Diversity and Structure of Eight Populations of Nerita yoldii along the Coast of China Based on Mitochondrial COI Gene
Source: Animals (Basel). 2024 Feb 25;14(5):718. doi: 10.3390/ani14050718 (PMC10930862; doi:10.3390/ani14050718)
Supplement: Supplementary file 1 [file animals-14-00718-s001.zip › animals-2720347-supplementary.pdf]

Table S1. The 34 haplotypes distribution of eight *N. yoldii* populations based on *COI* gene.

haplotype	SS	LH	XS	TZ	FD	XP	XM	ST	Overall
Hap_1	22	29	20	18	18	14	12	14	147
Hap_2	1								1
Hap_3	1								1
Hap_4	3	2	1	2	1	4	2		15
Hap_5	1	1	1	2	2	1			8
Hap_6	1						2	1	4
Hap_7	1		1	6	2	7	1		18
Hap_8	1		1			1			3
Hap_9	1								1
Hap_10		1						1	2
Hap_11			4						4
Hap_12			1						1
Hap_13			1						1
Hap_14				1					1
Hap_15				1					1
Hap_16					1				1
Hap_17					1				1
Hap_18					1			1	2
Hap_19						1		2	3
Hap_20						1			1
Hap_21						1			1
Hap_22							1	1	2
Hap_23							1		1
Hap_24							1		1
Hap_25							1		1
Hap_26							1		1
Hap_27							1		1
Hap_28							1		1
Hap_29								1	1
Hap_30								1	1
Hap_31								3	3
Hap_32								1	1
Hap_33								1	1
Hap_34								1	1
Total	32	33	30	30	26	30	24	28	233

Table S2. Genetic distance of eight *N. yoldii* populations based on *COI* gene.

population	SS	LH	XS	TZ	FD	XP	XM	ST
SS	0.0020							
LH	0.0014	0.0008						
XS	0.0023	0.0018	0.0026					
TZ	0.0027	0.0022	0.0030	0.0032				
FD	0.0019	0.0014	0.0023	0.0026	0.0020			
XP	0.0031	0.0027	0.0033	0.0034	0.0030	0.0037		
XM	0.0024	0.0019	0.0027	0.0031	0.0024	0.0035	0.0029	
ST	0.0025	0.0020	0.0028	0.0032	0.0025	0.0036	0.0029	0.0029

[S2] The diagonal is the distance within the population, and below the diagonal is the distance between populations.
